# Self-perceived oral health among Brazilian university students: a cross-sectional study

**DOI:** 10.1590/1807-3107bor-2024.vol38.0058

**Published:** 2024-12-09

**Authors:** Luana Beliago de Azevedo Costa, Rafaela de Oliveira Cunha, Isabel Cristina Gonçalves Leite

**Affiliations:** a Universidade Federal de Juiz de Fora – UFJF, School of Medicine, Department of Collective Health, Juiz de Fora, MG, Brazil.; b Universidade Federal de Juiz de Fora – UFJF, School of Medicine, Department of Collective Health, Juiz de Fora, MG, Brazil.

**Keywords:** Self Concept, Oral Health

## Abstract

This cross-sectional study aimed to analyze the self-perceived oral health of young university students at the Federal University of Juiz de Fora and identify the associated factors. Data were collected in 2021 using a self-administered questionnaire containing questions on students’ sociodemographic variables and oral health. Binary logistic regression was applied in the multivariate analysis using the SPSS (Statistical Package for the Social Sciences) software (version 20.0) for Windows. The final analysis included 1,316 students aged 17–24 years. The prevalence of negative self-perception of oral health was 14.1% (95%CI: 12.2–16.0). The following variables were associated with negative self-perception of oral health: single marital status (OR = 0.34; 95%CI: 0.12–0.98), monthly family income of up to three minimum wages (OR = 2.02; 95%CI: 1.32–3.09), non-regular use of dental services (OR = 2.29; 95%CI: 1.48–3.53), dissatisfaction with the last service (OR = 1.97; 95%CI: 1.23–3.16), fear of dental treatment (OR = 1.56; 95%CI: 1.06–2.29), dissatisfaction with the appearance of teeth and mouth (OR = 5.27; 95%CI: 3.37–8.22), and perceived need for dental treatment (OR = 6.94; 95%CI:3.14–15.33). In conclusion, most young university students had a positive self-perception of oral health. However, factors related to socioeconomic profile, access to oral health services, and satisfaction with one's appearance were found to increase the likelihood of having a negative self-perception of oral health.

## Introduction

Self-perception of oral health is a subjective indicator of morbidity and mortality that has multidimensional influences such as clinical and psychosocial issues.^1,2^ It encompasses an individual's subjective view of their social, functional, and psychological well-being associated with their oral health.^3,4^ Furthermore, it is associated with an individual's quality of life.^5^

Therefore, knowledge of this indicator and its associated conditions is crucial for the planning and implementation of public actions and policies in oral health to recognize the subjectivity involved in the health-disease process and address individual factors associated with quality of life.^6^

Considering this, several studies have been conducted with the objective of identifying the characteristics of the Brazilian population in relation to self-perception of oral health and its associated factors. Variables such as income, gender, race, age, tooth aesthetics, evaluation of the last visit, perceived need for dental treatment, reason for the last visit, non-health course, dental fear, family income, lower parental education, and use of public health services were associated with a negative self-perception of oral health in several studies among the Brazilian population, including different age groups.^6-14^ However, few studies have been conducted specifically with the college students, which has unique characteristics owing to their stage of life and particular socioeconomic and demographic profiles.^15^ Oral health and dental aesthetics impact psychological well-being of individuals. Facial characteristics and appearance are particularly relevant for adolescents and young adults. Therefore, it affects their self-perception, self-esteem, and quality of life,^16^ including their mental health.^17^ Thus, a negative self-perception of oral health becomes a barrier to social inclusion.^18^ Additionally, this population has characteristics associated with a higher probability of inadequate oral health and negative self-perception of oral health, such as accessing dental services out of necessity instead of prevention,^19^ deficient oral health knowledge,^20^ and consumption of cariogenic foods and beverages.^10^ Another study emphasized that the search for healthcare services was associated with students’ income, with higher income implying greater access to healthcare services.^15^

Therefore, the present study aimed to explore the self-perception of oral health among undergraduate students at the Federal University of Juiz de Fora (UFJF) and identify the associated socioeconomic, demographic, and oral health-related factors to determine the vulnerabilities related to this subjective indicator in this population.

## Methods

### Study design and participants

This cross-sectional census study included university students aged between 17 and 24 years from various face-to-face undergraduate courses at the Federal University of Juiz de Fora (UFJF) in 2021. It is a public institution for higher education headquartered in Juiz de Fora and an advanced field in Governador Valadares (MG). In 2021, 16,068 students were enrolled.

The study population included young university students enrolled in face-to-face undergraduate courses and aged between 17 and 24 years. The age group was selected based on the World Health Organization's (WHO) age classification of young individuals.^21^ Individuals who did not respond to the research questionnaire sent via e-mail despite three attempts (once a month) to contact them were considered sample loss. The Human Research Ethics Committee of the Federal University of Juiz de Fora (UFJF) approved this study (opinion number: 4,617,665).

### Data collection

The present study used data from a previous study. Cunha and Leite have described the formulation of the questionnaire in the digital format and the process of data collection.^19^ The questionnaire was created on Google Forms and sent via e-mail to all enrolled students, requiring their informed consent to access the free questionnaire.

A pilot study was conducted to analyze the potential difficulties in interpreting the questionnaire by the students. It included a group of 23 university students who answered the questions and provided critical feedback, suggestions, or possible doubts about them, thereby allowing authors to make necessary modifications.

The self-administered questionnaire was divided into three blocks and included questions related to socioeconomic and demographic characteristics, their undergraduate course, and the use of dental services and oral health conditions.

### Variables

The dependent variable investigated in this study was self-perception of oral health, a categorical variable inquired through the question "How would you describe the health of your teeth and mouth?" The answer was dichotomized between positive (excellent, very good, and good) and negative (fair and poor) based on several previous studies.^9,11,12,22^

The independent variables were selected based on previous studies ^6,8,9,14,16,22-24^ and grouped into two blocks: socioeconomic and demographic factors (Block 1) and factors associated with oral health (Block 2), as shown in Figure.

**Figure:**
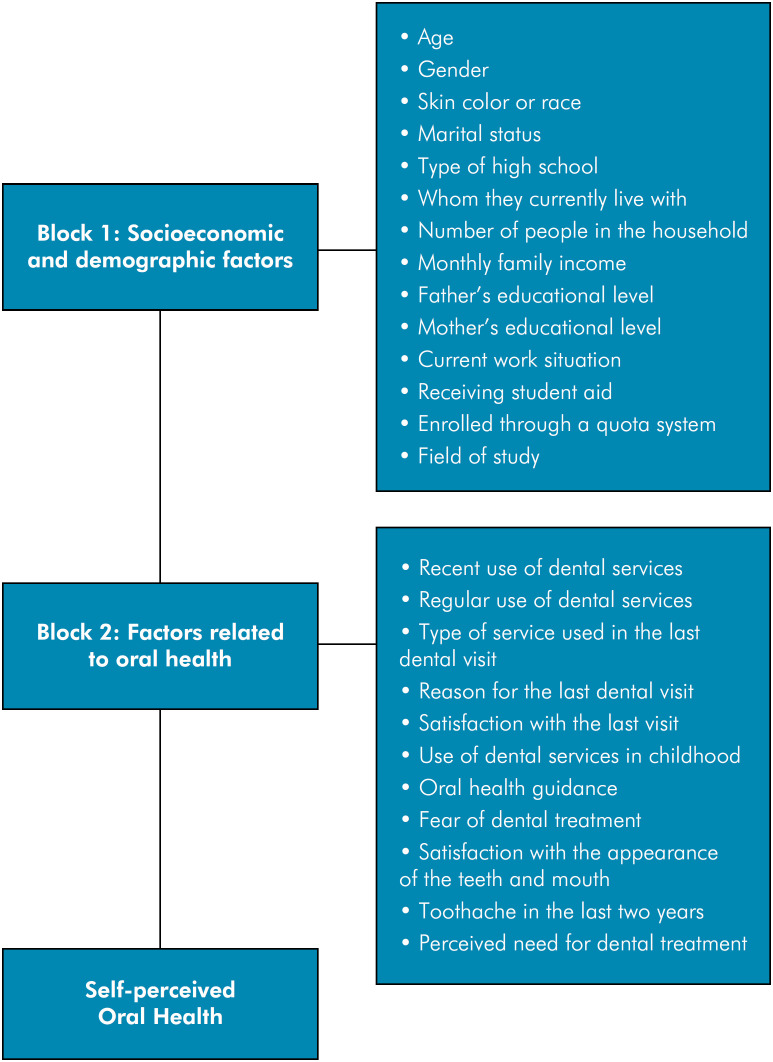
Theoretical model for analyzing the association of independent variables with self-perceived oral health by blocks.

Figure describes the formats in which the independent variables were collected and the categories for statistical analysis. Notably, the variable "current work situation" was relative to the undergraduate student and the variable "oral health guidance" was based on whether students received information about oral health during their life (Chat 1).

### Data analysis

The SPSS (Statistical Package for the Social Sciences) version 20.0 for Windows was used for data analysis. First, descriptive analyses were performed using absolute and relative frequencies. The association between dependent and independent variables was determined using bivariate analyses and logistic regression to estimate the crude and adjusted ORs, adhering to the 95% confidence interval. The independent variables associated with a p-value of ≤ 0.05 were included in the multiple models, and the variables with a p-value of < 0.05 were included in the final model.

## Results

Of the 16,068 students enrolled in 2021, 1,876 students responded to the questionnaire, corresponding to a response rate of 11.7%. Of these, 1,316 undergraduates met the inclusion criteria and comprised the final sample. According to data from the local academic record, these undergraduates had sociodemographic characteristics similar to those of freshmen at the institution. Regarding their socioeconomic and demographic profiles, there was a predominance of cisgender females aged between 20 and 24 years, self-declared white, single, coming from public schools, living with family members, sharing a household with three or four people, and having a family income of up to three minimum wages. Additionally, 75.4% were unemployed, 20.7% received student aid, 48.6% entered through quotas, and 62.6% studied subjects other than biological sciences or health.

Regarding the variables associated with oral health, it was observed that most students sought dental care recently (in the last two years), did not use dental services regularly, and used the private health system in the last visit, mostly out of necessity. Furthermore, it was highlighted that 43.5% were dissatisfied with the appearance of their teeth and mouth, 49.3% had a toothache in the last 2 years, and 63.8% perceived the need for dental treatment.

Considering the dependent variable in this study, self-perception of oral health, 14.1% (95%CI: 12.2–16.0) had a negative self-perception of their oral health. Table 1 presents the frequency distributions of the independent variables divided into blocks according to the outcome investigated.

**Table 1:** Participants’ socioeconomic, demographic, and oral health-related variables distributed according to self-perceived oral health.

Variable		Self-perceived oral health			
		Positive		Negative	
		n	%	n	%
**Block 1: Socioeconomic and demographic variables**					
Age					
	17 to 19 years	68	13.2	448	86.8
	20 to 24 years	117	14.6	68.3	85.4
Gender					
	Cisgender female	136	14.4	811	85.6
	Cisgender male	42	12	308	88
	Transgender/nonbinary/agender	7	36.8	12	63.2
Self-declared color					
	Whites	88	11.2	699	88.8
	Nonwhites	97	18.3	432	81.7
Marital status					
	Single	175	13.5	1119	86.5
	Married or stable union	10	45.5	12	84.5
Type of high school attended					
	Private	139	18.9	595	81.1
	Public	46	7.9	536	92.1
Whom do you currently live with?					
	Alone	16	13.1	106	86.9
	With friends or colleagues or spouse/partner/boyfriend or girlfriend	42	15.8	224	84.4
	Relatives	127	13.7	801	86.3
Number of people in the household					
	One or two	50	13.6	318	86.4
	Three or Four	114	14.9	652	85.1
	Five or more	21	11.5	161	88.5
Monthly family income					
	Up to three times the minimum wage	147	20.6	568	79.4
	More than three times the minimum wage	38	6.3	563	93.7
Father's educational level*					
	Did not attend school/incomplete elementary school	59	21.9	211	78.1
	Completed elementary school/incomplete high school	26	17.3	124	82.7
	Completed high school /incomplete higher education	53	11.5	407	88.5
	Completed higher education /postgraduate degree	31	8.2	347	91.8
Mother's educational level*					
	Did not attend school/incomplete elementary school	40	22.7	136	77.3
	Completed elementary school/incomplete high school	29	22.5	100	77.5
	Completed high school/incomplete higher education	70	14.6	409	85.4
	Completed higher education/postgraduate degree	46	8.8	478	91.2
Current work situation					
	Works	47	15.8	251	84.2
	Does not work	138	13.6	880	86.4
Receiving student aid					
	Yes	55	20.2	217	79.8
	No	130	12.5	914	87.5
Enrolled through a quota system					
	Yes	128	20	512	80
	No	57	8.4	619	91.6
Field of study					
	Biological or health sciences	51	10.4	441	89.6
	Others	134	16.3	690	83.7
**Block 2: Factors related to oral health**					
Recent use of dental services					
	Yes	128	12	938	88
	No	57	22.8	193	77.2
Regular use of dental services					
	Nonregular use	151	20.3	592	79.7
	Regular use	34	5.9	539	94.1
Type of service used in the last dental visit*					
	Public	54	28.1	138	71.9
	Private	127	11.3	992	88.7
Reason for the last dental visit*					
	Need	113	16.5	570	83.5
	Prevention	68	10.8	560	89.2
Satisfaction with the last visit*					
	Dissatisfied	43	3.1	87	66.9
	Satisfied	138	11.7	1043	88.3
Use of dental services in childhood					
	No	108	19	460	81
	Yes	77	10.3	671	89.7
Oral health guidance					
	No	32	34	62	66
	Yes	153	12.5	1069	87.5
Fear of dental treatment					
	Yes	71	22.8	240	77.2
	No	114	11.3	891	88.7
Satisfaction with the appearance of the teeth and mouth					
	Dissatisfied	157	27.4	416	72.6
	Satisfied	28	3.8	715	96.2
Toothache in the last two years					
	Yes	128	19.7	521	80.3
	No	57	8.5	610	91.5
Perceived need for dental treatment					
	Yes	178	21.2	662	78.2
	No	7	1.5	469	98.5

* Missing data were excluded.

Source: Research data.

* Missing data were excluded.

The variables of block 1, including gender, self-declared color, marital status, type of high school attended, monthly family income, paternal schooling, maternal schooling, receipt of student aid, admission by quotas, and knowledge area of the course, were associated with self-perception of oral health in the crude analysis. Considering block 2, all variables were associated with the outcome. After block-adjusted analysis, the following variables remained significant: gender, marital status, monthly family income, knowledge area of the course, regular use of dental services, type of service used in the last dental visit, satisfaction with the last appointment, fear of dental treatment, satisfaction with the appearance of teeth and mouth, toothache in the last two years, and perceived need for dental treatment.

In the final model, the following variables were associated with negative self-perception of oral health: single marital status, low monthly family income, irregular use of dental services, dissatisfaction with the last visit, fear of dental treatment, dissatisfaction with the appearance of their teeth and mouth, and perceived need for dental treatment. Tables 2 and 3 show the crude, block-adjusted ORs, and final model for negative self-perceived oral health.

**Table 2:** Association between self-perceived oral health and socioeconomic, demographic, and oral health-related variables.

Variable		Crude OR (IC 95%)	p*	Block-adjusted OR (IC 95%)	p **
**Block 1: Socioeconomic and demographic variables**					
Age			0,516		
	17 to 19 years	0,89 (0,64-1,22)			
	20 to 24 years	1			
Gender			0,9		0,021
	Cisgender female	0,29 (0,11-0,74)		3,47 (1,19-10,13)	
	Cisgender male	0,23 (0,09-0,63)		3,71 (1,22-11,27)	
	Transgender/nonbinary/agender	1		1	
Self-declared color			<0,001		0,310
	Whites	0,56 (0,41-0,77)		0,83 (0,56-1,19)	
	Nonwhites	1		1	
Marital status			< 0,001		0,001
	Single	0,19 (0,08-0,44)		0,19 (0,07-0,50)	
	Married or stable union	1		1	
Type of high school			<0,001		0,633
	Public	2,72 (1,91-3,87)		1,19 (0,58-2,44)	
	Private	1		1	
Whom they currently live with			0,652		
	Alone	0,95 (0,54-1,66)			
	With friends or colleagues or spouse/partner/boyfriend or girlfriend	1,18 (0,81-1,73)			
	Relatives	1			
Number of people in the household			0,483		
	One or two	1,20 (0,70-2,08)			
	Three or Four	1,34 (0,82-2,20)			
	Five or more	1			
Monthly family income			<0,001		<0,001
	Up to three times the minimum wage	3,83 (2,63-5,58)		2,55 (1,63-4,01)	
	More than three times the minimum wage	1		1	
Father's educational level			<0,001		0,248
	Did not attend school/incomplete elementary school	3,13 (1,96-4,99)		0,71 (0,40-1,26)	
	Completed elementary school/incomplete high school	2,35 (1,34-4,11)		0,86 (0,46-1,62)	
	Completed high school /incomplete higher education	1,46 (0,91-2,32)		1,02 (0,62-1,70)	
	Completed higher education/postgraduate degree	1		1	
Mother's educational level			<0,001		0,408
	Did not attend school/incomplete elementary school	3,06 (1,92-4,86)		0,88 (0,50-1,56)	
	Completed elementary school/incomplete high school	3,01 (1,80-5,03)		0,77 (0,42-1,42)	
	Completed high school/incomplete higher education	1,78 (1,20-2,64)		0,98 (0,62-1,55)	
	Completed higher education/postgraduate degree	1		1	
Current work situation			0,333		
	Works	1,19 (0,83-1,71)			
	Does not work	1			
Receiving student aid			0,001		0,441
	Yes	1,78 (1,26-2,52)		0,85 (0,57-1,28)	
	No	1		1	
Enrolled through a quota system			<0,001		0,203
	Yes	2,71 (1,94-3,79)		1,56 (0,79-3,11)	
	No	1		1	
Field of study			0,003		0,021
	Biological or health sciences	0,59 (0,42-0,84)		0,64 (0,44-0,94)	
	Others	1		1	
**Block 2: Factors related to oral health**					
Recent use of dental services			<0,001		0,365
	Yes	0,46 (0,33-0,65)		0,82 (0,54-1,26)	
	No	1		1	
Regular use of dental services			<0,001		<0,001
	Nonregular use	4,04 (2,74-5,97)		2,41 (1,55-3,75)	
	Regular use	1		1	
Type of service used in the last dental visit			<0,001		0,021
	Public	3,06 (2,12-4,40)		1,65 (1,08-2,52)	
	Private	1		1	
Reason for the last dental visit			0,003		0,357
	Need	1,63 (1,18-2,25)		0,83 (0,57-1,22)	
	Prevention	1		1	
Satisfaction with the last visit			<0,001		0,007
	Dissatisfied	3,74 (2,49-5,61)		1,92 (1,19-3,08)	
	Satisfied	1		1	
Use of dental services in childhood			<0,001		0,129
	No	2,05 (1,49-2,80)		1,33 (0,92-1,92)	
	Yes	1		1	
Oral health guidance			<0,001		0,056
	No	3,61 (2,28-5,71)		1,72 (0,98-3,02)	
	Yes	1		1	
Fear of dental treatment			<0,001		0,033
	Yes	2,31 (1,64-3,21)		1,52 (1,03-2,23)	
	No	1		1	
Satisfaction with the appearance of the teeth and mouth			<0,001		<0,001
	Dissatisfied	9,64 (6,33-14,66)		5,29 (3,39-8,23)	
	Satisfied	1		1	
Toothache in the last two years			<0,001		0,028
	Yes	2,63 (1,88-3,67)		1,54 (1,05-2,26)	
	No	1		1	
Perceived need for dental treatment			<0,001		<0,001
	Yes	18,01 (8,39-38,69)		7,73 (3,50-17,05)	
	No	1		1	

OR: Odds ratio; CI: Confidence interval.

* Pearson's chi-square test

** Binary logistic regression.

**Table 3:** Final model of variables associated with self-perceived oral health among university students.

Variable		Adjusted OR p **	Final model (IC 95%)
**Block 1: Socioeconomic and demographic variables**			
Gender			0,263
	Cisgender female	1,88 (0,62-5,68)	
	Cisgender male	1,82 (0,58-5,72)	
	Transgender/nonbinary/agender	1	
Marital status			0,046
	Single	0,34 (0,12-0,98)	
	Married or stable union	1	
Monthly family income			0,001
	Up to three times the minimum wage	2,02 (1,32-3,09)	
	More than three times the minimum wage	1	
Field of study			0,171
	Biological or health sciences	0,75 (0,50-1,13)	
	Others	1	
**Block 2: Factors related to oral health**			
Regular use of dental services			<0,001
	Nonregular use	2,29 (1,48-3,53)	
	Regular use	1	
Type of service used in the last dental visit			0,070
	Public	1,48 (0,97-2,28)	
	Private	1	
Satisfaction with the last visit			0,005
	Dissatisfied	1,97 (1,23-3,16)	
	Satisfied	1	
Fear of dental treatment			0,025
	Yes	1,56 (1,06-2,29)	
	No	1	
Satisfaction with the appearance of the teeth and mouth			0,001
	Dissatisfied	5,28 (3,37-8,22)	
	Satisfied	1	
Toothache in the last two years			0,080
	Yes	1,41 (0,96-2,07)	
	No	1	
Perceived need for dental treatment			<0,001
	Yes	6,94 (3,14-15,33)	
	No	1	

OR: Odds ratio; CI: Confidence interval.

** Binary logistic regression.

**Table:** Chart. Categories for collecting and analyzing the study's independent variables.

Variable	Collection Categories	Categories for analysis
Age	Uncategorized	17 to 19 years old; 20 to 24 years old
Gender	Cisgender female; cisgender male; transgender female; agender; and non-binary.	Cisgender female; cisgender male; transgender/non-binary/agender.
Skin color or race	White; black; brown; yellow and indigenous.	Whites; non-whites.
Marital status	Married or in a stable union; single; separated or divorced and widowed.	Single; married or in a stable union.
Type of school that attended high school	All in public schools; all in private schools; most in public schools; most in private schools.	Public school; private school.
Who you currently live with	Alone; with parents and/or other relatives; with friends or colleagues; with a spouse/partner/boyfriend/girlfriend.	Alone; with friends or colleagues or Spouse/partner/boyfriend/girlfriend; family.
Number of people in the household	Only me; two; three; four; five or more.	One or two; three or four; five or more.
Monthly household income	Up to 1 minimum wage; from 1 to 1.5 minimum wage; from 1.5 to 3 minimum wages; from 3 to 4.5 minimum wages; from 4.5 to 6 minimum wages; from 6 to 10 minimum wages; from 10 to 30 minimum wages; and above 30 minimum wages.	Up to 3 minimum wages; above 3 minimum wages.
Paternal schooling	He did not study; incomplete elementary school; complete elementary school; incomplete high school; complete high school; incomplete higher education; complete higher education; Graduate; He doesn't know how to inform.	Not studied/incomplete elementary school; complete elementary school/incomplete high school; complete high school / incomplete higher education; Complete Higher Education/Post-Graduation.
Maternal schooling	He did not study; incomplete elementary school; complete elementary school; incomplete high school; complete high school; incomplete higher education; complete higher education; Graduate; He doesn't know how to inform.	Not studied/incomplete elementary school; complete elementary school/incomplete high school; complete high school / incomplete higher education; Complete higher education/post-graduation.
Current working situation	Works; it doesn't work.	Yes; No
Receiving Student Aid	Yes; No.	Yes; No.
Quota admission	No; yes; by ethnic-racial criteria; Yes; by income criterion; Yes; for having studied in a public or private school with a scholarship; Yes; by a system that combines two or more of the above criteria.	No (for the answer "no"); yes (for the answers "yes; by ethnic-racial criteria"; "yes; by income criterion"; "Yes; for having studied in a public or private school with a scholarship"; "Yes; by a system that combines two or more of the above criteria").
Course Knowledge Area	Exact and earth sciences; biological sciences; Engineering; health sciences; agricultural sciences; applied social sciences; Humanities; and linguistics; literature and arts.	Biological or health sciences; Other.
Recent use of dental services	Yes; No	Yes; No
Regular use of dental services	I never go to the dentist; I go to the dentist when I have a problem or when I know I need to have something tidy; I go to the dentist occasionally; whether or not I have some kind of problem; I go to the dentist regularly.	I don't use it regularly (I never go to the dentist; I go to the dentist when I have a problem or when I know I need to have something tidy); Regular use (I go to the dentist occasionally; whether I have a problem or not; I go to the dentist regularly).
Type of service used in the last query	Public service (health center; dental specialty center; dental emergency care units; college or educational institution in the area of Dentistry) and private practice or health plan	Public; private.
Reason for last query	Dental urgency - cases of pain; gingival bleeding; tooth decay; need for root canal treatment; extraction or replacement of restoration; Review/ Prevention/ Checkup/ Routine/ Cleaning.	Need (for the answer "dental urgency - cases of pain; gingival bleeding; dental caries; need for root canal treatment; extraction or replacement of restoration"); Prevention (Review/Prevention/Checkup/Routine/Cleaning).
Satisfaction with the last call	Very good; good; regular; bad; Very bad.	Satisfied (for the answer "very good" and "good"); dissatisfied (for the answers "fair"; "bad" and "very bad").
Use of dental services in childhood	I never went to the dentist when I was a kid; I went to the dentist a few times when I was a kid; I went to the dentist many times when I was a child; I don't know.	No (for the answers "I never went to the dentist when I was a child"; "I went to the dentist a few times when I was a child" and "I don't know"); Yes (for "I went to the dentist many times when I was a kid").
Oral Health Guidelines	Yes; No.	Yes; No.
Fear of treatment	Yes; No.	Yes; No.
Satisfaction with the appearance of teeth and mouth	Very satisfied; satisfied; neither satisfied nor dissatisfied; unsatisfied; Very dissatisfied.	Satisfied (for the answers "Very satisfied" and "satisfied"); dissatisfied (for "neither satisfied nor dissatisfied"; "dissatisfied"; "very dissatisfied".
Toothache in the last 2 years	Yes; No.	Yes; No.
Perceived need for dental treatment	Yes; No.	Yes; No.

Source: Prepared by the author (2022).

## Discussion

Oral health conditions and self-perception are influenced by social determinants of health.^25,26^ Therefore, it is essential to understand these factors to ensure equity and reduce inequalities in provision of oral healthcare.^27^

In the present study, the prevalence of negative self-perception of oral health was 14.1%. It was associated with the following variables: single marital status, low monthly family income, irregular use of dental services, dissatisfaction with the last appointment, fear of dental treatment, dissatisfaction with the appearance of teeth and mouth, and perceived need for dental treatment. The present study results were consistent with the scientific literature that indicates a predominance of positive self-perception in this age group. Data from the National Oral Health Survey (PNS) identified a higher prevalence of negative self-perception in individuals aged 15–19 years and 18 years or older, with an important regional disparity between the North/Northeast and South/Southeast regions, with the latter having a lower prevalence.^6,24,28^ It is essential to emphasize that although these studies involved the age group included in the present study, they did not focus on the young university population and partially involved them or included them in a broader age group. Thus, they may overestimate or underestimate the results. They may ignore the social context of the university since individuals who have access to universities may represent, even today, a portion of the socioeconomically privileged population despite affirmative action policies that have increased access to universities.^11,29^

Additionally, analyses involving the university population highlighted a predominance of positive self-perception of oral health, despite the prevalence of negative self-perception being higher than that identified in this study.^11,22^ However, these studies included a wider age range, which may explain the difference in findings, as the prevalence of positive self-perception was higher among younger people.^30^

The socioeconomic variables that were associated with self-perceived oral health following adjustments in analysis were marital status and monthly family income. Regarding marital status, single status was associated with a lower frequency of negative self-perception of oral health. This variable was not identified in studies involving the young population but was associated with older adults in another study.^13^ Doctors seem to be more concerned with the appearance of patients’ teeth and oral health and seek to maintain self-care. These factors directly influence the self-esteem and social lives of these individuals, as identified by several authors.^16, 31, 32^ Regarding monthly family income, the present study identified that individuals with an income of up to three minimum wages were 102% more likely to have a negative outcome than individuals with higher income. This finding was consistent with a previous study.^22^ Other contextual or individual variables related to economic issues that have been associated with negative self-perception of oral health in previous studies include lower per capita income, lower social class, or lower socioeconomic status.^23,30^ Access to dental goods and services and oral health education is probably a major differential in the population with greater purchasing power. Individuals with a high income have easier access to oral health goods and services^8,33^ and oral health information.^34^ Subsequently, they are more likely to ensure self-care and satisfaction with the appearance of their teeth and mouth, a variable that was strongly associated with self-perceived oral health in the present study.

Considering the factors associated with oral health, the following variables were associated with the outcome after the adjusted analysis: regular use of dental services, satisfaction with the last visit, fear of dental treatment, satisfaction with the appearance of teeth and mouth, and perceived need for dental treatment. Irregular use of dental services was associated with a higher frequency of negative self-perception. This result is similar to that described by Escheverria,^12^ who found that the regular use of dental services was associated with a greater chance of having a positive self-perception of oral health. The regular use of these services helps in the maintenance of oral health and early diagnosis of health conditions, becoming a predictor of satisfactory oral health. Previous research involving university population has identified that they used dental services when needed instead of on a regular basis.^19^ This highlights the need to pay attention to the mechanisms that increase the access of these individuals to oral health services. These mechanisms may involve the development of public policies for prevention and health promotion focused on achieving the interests of this population and including them in oral healthcare programs.

Consistent with the results of a study conducted using data from the 2019 National Health Survey (PNS) involving 60,202 adults aged 18 years and above,^9^ satisfaction with the last visit was also associated with a lower frequency of negative self-perception of oral health in the present study. Gonçalves^35^ identified that dissatisfaction with a service was strongly associated with the non-resolution of users’ needs. A lack of resolution gives users the perception that their oral health remains problematic. Situations such as lack of instructions about oral healthcare, insufficient consultation time, lack of important medical records, non-attendance in the afternoon shift by the Oral Health Team (OHT), time incompatible with users’ needs, and not looking for the user in case of treatment drop out were associated with greater dissatisfaction with care in Basic Health Units.^36^ These data highlight the need for primary care professionals to improve access and care for users.

Reportedly, fear was associated with a greater chance of negative self-perception of oral health. The prevalence of fear of dental care was 23.6%, similar to that of a previous study involving university students.^11^ Fear leads people to postpone dental appointments for as long as possible and seek care only in cases of extreme distress. Thus, it becomes a barrier to the regular use of services, thereby aggravating health problems and affecting subjective conditions.^37^

Another variable that was associated with the outcome was satisfaction with the appearance of teeth and mouth. In this study, dissatisfaction with the appearance of teeth and mouth was related to a 4-fold increase in the likelihood of having a negative self-perception of oral health. Militi^16^ indicated the influence of dental appearance and conditions related to facial features and physiognomy on the self-perception of oral health among young adults. Other studies have demonstrated the association between self-perception of oral health, perception of self-esteem, and quality of life in this population, providing evidence for the influence of these variables on emotional well-being and social relationships.^16,17,31,32^ These results emphasize the importance of dental appearance in the judgment of individuals regarding their oral health. Furthermore, considering the impact of these issues on their lives, it highlights the need to consider aesthetic concerns in the procedures and services offered by the healthcare system.^38^

The perceived need for dental treatment was also strongly associated with negative self-perception of oral health. Probably, individuals who realize that they need dental treatment believe that their oral health is unsatisfactory. This problem occurs when users do not develop the capacity to perceive their needs due to a lack of knowledge and/or access to oral health education. Some studies have shown an association between a lack of access to oral health information, failure to self-perceive problems, and the search for access to oral health services.^39,40^ This variable was also associated with the self-perception of oral health by Rebouças.^7^

Regarding socioeconomic and demographic factors, other studies have indicated that male gender, non-white skin color, non-health areas of knowledge, lower parental education, and older age are associated with a higher probability of negative self-perception of oral health.^6,14,22,24^ However, the present study did not find a statistically significant association between these variables and the outcome. Age may not have been associated with the self-perception of oral health in this study because of the small variability among the categories analyzed. Additionally, unlike previous studies that included wider age group, the present study only evaluated the young population. Based on previous studies, the reasons for the last dental visit and the type of health service were also associated with self-perception of oral health.^9, 23^

These results indicate that self-perception of oral health is influenced by factors that are directly or indirectly associated with access to dental goods and services, whether due to income, regular use, satisfaction with the services, or fear that prevents them from accessing them. Additionally, it is influenced by issues involving appearance. Furthermore, marital status may be a variable that indirectly affects this concern.

This study had limitations. Since it was a cross-sectional study, it was not possible to determine a cause-and-effect relationship. Additionally, the questionnaire used for data collection depended on self-reported information, which does not guarantee the veracity of the facts. Furthermore, the motivation to participate in the study may influence the results, since the sample may be composed of individuals who place more importance on oral health.

This study contributes to the identification of factors that can influence negative self-perception of oral health, considering the scarcity of research on this indicator focusing on the university population despite its important influence on individuals’ lives. University students are a population with a specific socioeconomic, demographic, cultural, psychological, and oral health profile considering their age and the social context in which they live, which are especially influenced by situations that harm social inclusion and self-esteem. However, this population is still neglected in oral healthcare programs in the country.

## Conclusions

This study identified a 14.1% prevalence of negative self-perception among university students. The socioeconomic variables associated with negative self-perception of oral health were single marital status and family income of up to three minimum wages, while factors associated with oral health were non-regular use of dental services, dissatisfaction with the last dental visit, fear of dental treatment, dissatisfaction with the appearance of teeth and mouth, and perceived need for treatment.
